# Tractography passes the test: Results from the diffusion-simulated connectivity (disco) challenge

**DOI:** 10.1016/j.neuroimage.2023.120231

**Published:** 2023-06-16

**Authors:** Gabriel Girard, Jonathan Rafael-Patiño, Raphaël Truffet, Dogu Baran Aydogan, Nagesh Adluru, Veena A. Nair, Vivek Prabhakaran, Barbara B. Bendlin, Andrew L. Alexander, Sara Bosticardo, Ilaria Gabusi, Mario Ocampo-Pineda, Matteo Battocchio, Zuzana Piskorova, Pietro Bontempi, Simona Schiavi, Alessandro Daducci, Aleksandra Stafiej, Dominika Ciupek, Fabian Bogusz, Tomasz Pieciak, Matteo Frigo, Sara Sedlar, Samuel Deslauriers-Gauthier, Ivana Kojčić, Mauro Zucchelli, Hiba Laghrissi, Yang Ji, Rachid Deriche, Kurt G Schilling, Bennett A. Landman, Alberto Cacciola, Gianpaolo Antonio Basile, Salvatore Bertino, Nancy Newlin, Praitayini Kanakaraj, Francois Rheault, Patryk Filipiak, Timothy M. Shepherd, Ying-Chia Lin, Dimitris G. Placantonakis, Fernando E. Boada, Steven H. Baete, Erick Hernández-Gutiérrez, Alonso Ramírez-Manzanares, Ricardo Coronado-Leija, Pablo Stack-Sánchez, Luis Concha, Maxime Descoteaux, Mansour L. Sina, Caio Seguin, Andrew Zalesky, Kenji Marshall, Erick J. Canales-Rodríguez, Ye Wu, Sahar Ahmad, Pew-Thian Yap, Antoine Théberge, Florence Gagnon, Frédéric Massi, Elda Fischi-Gomez, Rémy Gardier, Juan Luis Villarreal Haro, Marco Pizzolato, Emmanuel Caruyer, Jean-Philippe Thiran

**Affiliations:** a CIBM Center for Biomedical Imaging, Switzerland; b Radiology Department, Centre Hospitalier Universitaire Vaudois and University of Lausanne, Lausanne, Switzerland; c Signal Processing Laboratory (LTS5), École Polytechnique Fédérale de Lausanne (EPFL), Lausanne, Switzerland; d Univ Rennes, Inria, CNRS, Inserm, IRISA UMR 6074, Empenn ERL U-1228, Rennes, France; e A.I. Virtanen Institute for Molecular Sciences, University of Eastern Finland, Kuopio, Finland; f Department of Neuroscience and Biomedical Engineering, Aalto University, Espoo, Finland; g Department of Psychiatry, Helsinki University Hospital, Helsinki, Finland; h Waisman Center, University of Wisconsin-Madison, Madison, WI, United States; i Department of Radiology, University of Wisconsin-Madison, Madison, WI, United States; j Department of Medicine, University of Wisconsin-Madison, Madison, WI, United States; k Department of Medical Physics, University of Wisconsin-Madison, Madison, WI, United States; l Department of Psychiatry, University of Wisconsin-Madison, Madison, WI, United States; m Diffusion Imaging and Connectivity Estimation (DICE) Lab, Department of Computer Science, University of Verona, Verona, Italy; n Translational Imaging in Neurology (ThINk), Department of Biomedical Engineering, University Hospital Basel and University of Basel, Basel, Switzerland; o Department of Advanced Biomedical Sciences, University of Naples Federico II, Naples, Italy; p Sherbrooke Connectivity Imaging Laboratory (SCIL), Department of Computer Science, University of Sherbrooke, Sherbrooke, QC, Canada; q Brno Faculty of Electrical Engineering and Communication, Department of mathematics, University of Technology, Brno, Czech Republic; r Department of Neuroscience, Rehabilitation, Ophthalmology, Genetics, Maternal and Child Health (DINOGMI), University of Genoa, Genoa, Italy; s AGH University of Science and Technology, Kraków, Poland; t Sano Centre for Computational Personalised Medicine, Kraków, Poland; u Laboratorio de Procesado de Imagen (LPI), ETSI Telecomunicación, Universidad de Valladolid, Valladolid, Spain; v Athena Project Team, Centre Inria d’Université Côte d’Azur, France; w Department of Radiology and Radiological Sciences, Vanderbilt University Medical Center, Nashville, TN, United States; x Department of Electrical and Computer Engineering, Vanderbilt University, Nashville, TN, United States; y Brain Mapping Lab, Department of Biomedical, Dental Sciences and Morphological and Functional Images, University of Messina, Messina, Italy; z Center for Advanced Imaging Innovation and Research (CAI2R), Department of Radiology, NYU Langone Health, New York, NY, United States; aa Department of Neurosurgery, Perlmutter Cancer Center, Neuroscience Institute, Kimmel Center for Stem Cell Biology, NYU Langone Health, New York, NY, United States; ab Department of Radiology, Stanford University, Stanford, CA, United States; ac Computer Science Department, Centro de Investigación en Matemáticas A.C, Guanajuato, México; ad Instituto de Neurobiología, Universidad Nacional Autónoma de México, Juriquilla, Querétaro, México; ae Department of Biomedical Engineering, The University of Melbourne, Parkville, Victoria, Australia; af Melbourne Neuropsychiatry Centre, Department of Psychiatry, The University of Melbourne and Melbourne Health, Parkville, Victoria, Australia; ag School of Biomedical Engineering, The University of Sydney, Sydney, Australia; ah Department of Psychological and Brain Sciences, Indiana University, Bloomington IN, United States; ai McGill University, Montréal, QC, Canada; aj Department of Radiology and Biomedical Research Imaging Center (BRIC), The University of North Carolina at Chapel Hill, Chapel Hill, NC, United States; ak School of Computer Science and Engineering, Nanjing University of Science and Technology, Nanjing, China; al Department of Applied Mathematics and Computer Science, Technical University of Denmark, Kgs. Lyngby, Denmark; am Center for Complex Network Intelligence (CCNI), Tsinghua Laboratory of Brain and Intelligence (THBI), Tsinghua University, Beijing, China; an Department of Biomedical Engineering, Tsinghua University, Beijing, China; ao Institut de Biologie de Valrose, Université Côte d’Azur, Nice, France

**Keywords:** Diffusion MRI, Connectivity, Monte carlo simulation, Tractography, Numerical substrates, Microstructure, Challenge

## Abstract

Estimating structural connectivity from diffusion-weighted magnetic resonance imaging is a challenging task, partly due to the presence of false-positive connections and the misestimation of connection weights. Building on previous efforts, the MICCAI-CDMRI Diffusion-Simulated Connectivity (DiSCo) challenge was carried out to evaluate state-of-the-art connectivity methods using novel large-scale numerical phantoms. The diffusion signal for the phantoms was obtained from Monte Carlo simulations. The results of the challenge suggest that methods selected by the 14 teams participating in the challenge can provide high correlations between estimated and ground-truth connectivity weights, in complex numerical environments. Additionally, the methods used by the participating teams were able to accurately identify the binary connectivity of the numerical dataset. However, specific false positive and false negative connections were consistently estimated across all methods. Although the challenge dataset doesn’t capture the complexity of a real brain, it provided unique data with known macrostructure and microstructure ground-truth properties to facilitate the development of connectivity estimation methods.

## 1. Introduction

Over the last decade, protocols for diffusion-weighted magnetic resonance imaging (DW-MRI) acquisition, local modelling, tractography algorithms, and connectivity mapping methods have considerably improved (Jeurissen et al., 2017; Sotiropoulos and Zalesky, 2019; Sporns, 2011). However, concerns remain about the reliability of connectivity mapping. International tractography challenges (Côté et al., 2013; Fillard et al., 2011; Maffei et al., 2022; Maier-Hein et al., 2017; Nath et al., 2020) have shown limitations in the ability of tractography to correctly identify binary connectivity and identify white matter pathways consitently. In particular, Maier-Hein et al. (2017) showed that tractography may produce an abundance of false positive connections. Moreover, studies on animal models showed that, albeit tractography can correctly identify connections, the estimated connection weight does not always agree with *ex vivo* tracing data (Ambrosen et al., 2020a; Aydogan et al., 2018; Azadbakht et al., 2015; Delettre et al., 2019; Donahue et al., 2016; Essen et al., 2014; Girard et al., 2020; 2021; van den Heuvel et al., 2015; Jbabdi et al., 2013; Schilling et al., 2019a; Thomas et al., 2014). For instance, Donahue et al. (2016) reported the correlation between *ex vivo* tract tracing data and tractography estimation to be *r* = 0.59, on the intrahemispheric connections the monkey brain. Despite tract tracing being among the best available data to validate diffusion tractography, it is not possible to have the full ground-truth micro- and macro-structure on animal models.

The rich signal from physical MRI phantoms has been used to test and validate methods (Fillard et al., 2011; Schilling et al., 2019b), but their macrostructural complexity is insufficient for quantifying connectivity. Numerical phantoms have also been proposed and demonstrated to be important tools for methods development (Caruyer et al., 2014; Close et al., 2009; Neher et al., 2013), but their biological fidelity for microstructure is limited. Monte Carlo methods (Hall and Alexander, 2009; Lee et al., 2021; Rafael-Patino et al., 2020) can provide realistic microscopic DW-MRI signals, but they are generally limited to a single voxel signals or to a substrate of only a few voxels in size. Recently, Rafael-Patino et al. (2020) proposed a novel diffusion Monte Carlo simulator able to generate billions of particles. This allows for large-scale substrates with both microscopic and macroscopic complexity, suitable for structural connectivity validation.

The MICCAI-CDMRI 2021 Diffusion-Simulated Connectivity (DiSCo) challenge (Girard et al., 2021) was organized to compare structural connectivity estimation methods using three novel large-scale complex numerical phantoms designed for connectivity assessment (Rafael-Patino et al., 2021a; 2021b). Fourteen teams, adding up to 57 researchers, submitted 111 weighted connectivity matrices estimating the ground-truth connectivity. Results from the challenge are presented below.

## 2. Methods

### 2.1. Synthetic data

**Fig. 1.**
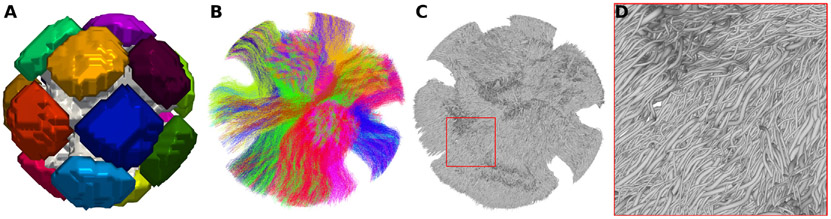
Ground-truth test dataset composed of 11,032 numerical tubular fibres. (A) 3D rendering showing the synthetic white matter mask (gray) and the 16 ROIs (colors). (B) Trajectories of the fibres of the 26 bundles, each shown using a different color. (C-D) 3D mesh of the outer layer of numerical fibres.

The three numerical phantoms (training, validation and test phantoms) used for the DiSCo challenge (Rafael-Patino et al., 2021b) are composed of approximately 12,000 numerical tubular fibres. The tubular fibres’ outer diameter ranges from 2.0 *μm* to 6 *μm*, sampled from a gamma distribution Γ(κ,θ), with shape, κ=0.5, and scale θ=0.007. The inner diameter of each fibre ranges from 1.4 *μm* to 4.2 *μm*, simulating a fixed g-ratio of 0.7 (Cercignani et al., 2017; Chomiak and Hu, 2009). The numerical fibres connect pairs of Regions of Interest (ROIs) among the 16 ROIs of each phantom (see Fig. 1A). No other numerical compartments were added to the substrates. For the three phantoms, the average percentage of connections with non-zero connection weight is 22.2% among all possible connections (120 pairs of ROIs). The connectivity weight between two ROIs was defined as the sum of the cross-sectional areas of fibres interconnecting the regions. The normalized connection weights range from 0.007 to 0.092 for the three phantoms, resulting with the smallest connection having 7.6% of the weight of the largest one. Those connection weights derive from the numerical phantom initialization parameters described in Rafael-Patino et al. (2021a). Fig. 1B, C, D show the ground-truth synthetic fibre trajectories of the test dataset where fibres are curved and intermingle with other fibres.

The simulation substrates have an unprecedented volume of 1 cubic millimeter, resulting in an image size of 40 × 40 × 40 voxels of 25 *μm* isotropic resolution. To the best of our knowledge, this is the largest image volume achieved for the Monte Carlo simulation of the DW-MRI signal in complex numerical substrates. Within each voxel, the signal was simulated using Monte Carlo simulations of spin dynamics with a density of one particle per cubic micrometer (Rafael-Patino et al., 2021a; Romascano et al., 2019). Rafael-Patino et al. (2020) showed this was a sufficient number of particles to obtain a robust estimation of the diffusion signal in complex fibre geometries. Particles initiated within the inner diameter of the fibres and outside the outer diameter of the fibres were used to generate the DW-MRI signal. The particles initiated between the outer and inner diameter (in myelin water) were discarded. The voxel-wise intratubular volume fraction reaches 52% in the central portion of the numerical phantoms (Rafael-Patino et al., 2021b). The mean voxel-wise fibre diameter is 2.25 *μm* with up to 82 tubular fibres per voxel and up to 5 distinct bundles (Rafael-Patino et al., 2021b).

The DW-MRI protocol is composed of 360 measurements, uniformly distributed over 4 b-value shells (1000, 1925, 3094, 13,191 *s/mm*^2^), as suggested in ActiveAx (Alexander et al., 2010; Daducci et al., 2015), and corrupted with Rician noise with signal-to-noise ratio of 30. The resulting DW-MRI signal is affected by the microscopic properties of the synthetic white matter, such as fibre diameter, packing densities, fibre dispersion and water diffusing around fibres, while also having targeted macroscopic properties like the smoothness of the trajectories and fibres organized in bundles.

### 2.2. Challenge task

Participating teams had access to one dataset for training, which included the noisy and noiseless DW-MRI signals, the fibre volume fraction map, the label map of the ROIs defining the connectivity endpoints, the synthetic fibre trajectories and their diameter, and the ground-truth connectivity matrix. Additionally, participants had access to one dataset for validation with the noisy DW-MRI signal, label map and ground-truth connectivity matrix. Participating teams were provided with the noisy DW-MRI signal and a label map (ROIs) of the test dataset, and were asked to submit a 16 × 16 weighted connectivity matrix. Participants were free to select any method to compute the matrix weights. The estimated connection weights between any two pairs of ROIs were compared with the ground-truth total cross-sectional area of the synthetic fibres connecting both ROIs. The teams had to select methods to obtain estimates of the cross-sectional area from their tractography results, such as the proportion or volume of streamlines, or microstructure properties or geometrical features estimated for bundles (Assaf et al., 2008; Daducci et al., 2014; Dimitriadis et al., 2017; Hagmann et al., 2008; 2007; Messaritaki et al., 2019; Smith et al., 2015; Sotiropoulos and Zalesky, 2019; Tournier et al., 2019; Yeh et al., 2021). Teams could submit up to ten connectivity matrices.

### 2.3. Connectivity evaluation

The Pearson correlation coefficient (*r*) between the ground-truth matrix and the submitted matrices was used for ranking the teams (Caminiti et al., 2021; Donahue et al., 2016). Moreover, the fraction of valid connectivity weight was computed to compare submissions (Côté et al., 2013; Maier-Hein et al., 2017). This fraction corresponds to the sum of the matrix weights in pairs of regions connected in the ground-truth connectivity matrix divided by the sum of all weights. A Receiver Operating Characteristic (ROC) analysis was also performed (Ambrosen et al., 2020b; Girard et al., 2020; Maffei et al., 2022; Schilling et al., 2019b; Thomas et al., 2014). The true positives (TP) and true negatives (TN) are connections correctly identified as connected and not connected in both the participant matrix and the ground-truth matrix, respectively. The false positives (FP) are connections wrongly identified as connected in the participant matrix. Similarly, the false negatives (FN) are connections erroneously identified as not connected. The ROC curves were constructed by iteratively thresholding the submitted connectivity matrices, starting with a threshold higher than the maximum, thus yielding no pair of ROIs connected, resulting in a specificity ($\frac{TN}{TN+FP}$) of 1 and sensitivity ($\frac{TP}{TP+FN}$) of 0 (all pairs of ROIs not connected in the ground-truth are correctly identified, no ROIs are identified as connected). The threshold is then iteratively reduced until all ROIs are identified as connected, producing a sensitivity of 1. The quicker the sensitivity rises to 1 while the specificity remains high, the better the binary connectivity classification performance of the method. The Area Under the ROC Curve (AUC) summarizes the plot with a number between 0 and 1. The AUC approaches 1 if there are few or no classification errors (a random connectivity matrix would yield an AUC of 0.5). Moreover, we studied the accuracy ($\frac{TP+TN}{TP+TN+FP+FN}$) of the submitted matrices using a threshold selected as 5% of their maximum value. This threshold was fixed following the connectivity weights of the ground-truth matrix.

## 3. Results

**Fig. 2.**
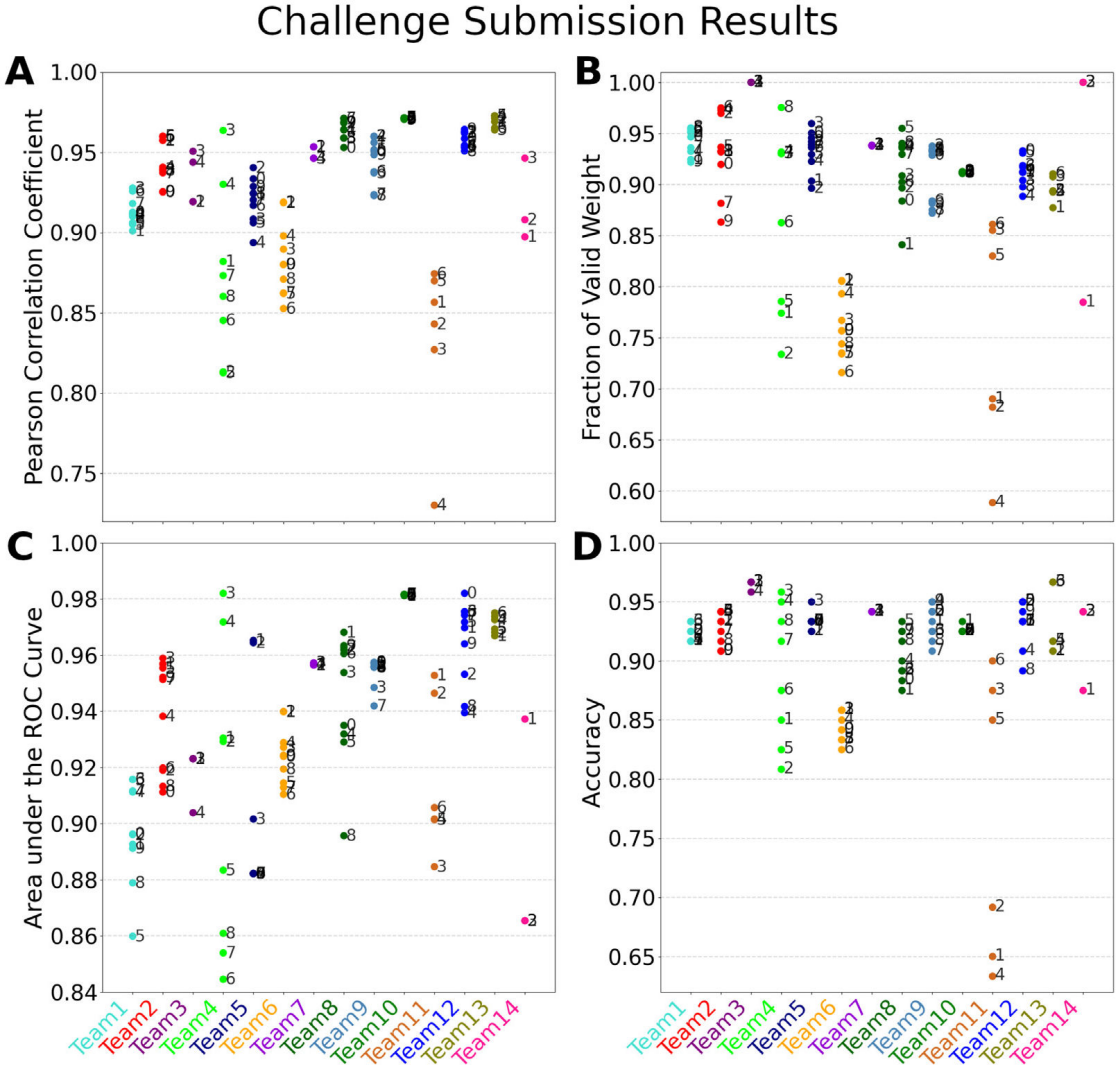
Challenge submission results of the 14 participating teams (111 submissions). (A) Fraction of valid connectivity weight in pairs of regions connected in the ground-truth connectivity matrix. (B) Pearson correlation coefficient between the participant’s submitted matrices and the ground-truth connectivity matrix of the validation dataset. (C) The area under the ROC curve (AUC) computed from the submitted matrices and the ground-truth binary connectivity matrix. (D) The accuracy (fraction of correctly identified pairs of ROIs, out of 120) of the binarised submitted matrices, thresholded at 5% of their maximal value. Numbers indicate the submission indices of each team.

**Fig. 3.**
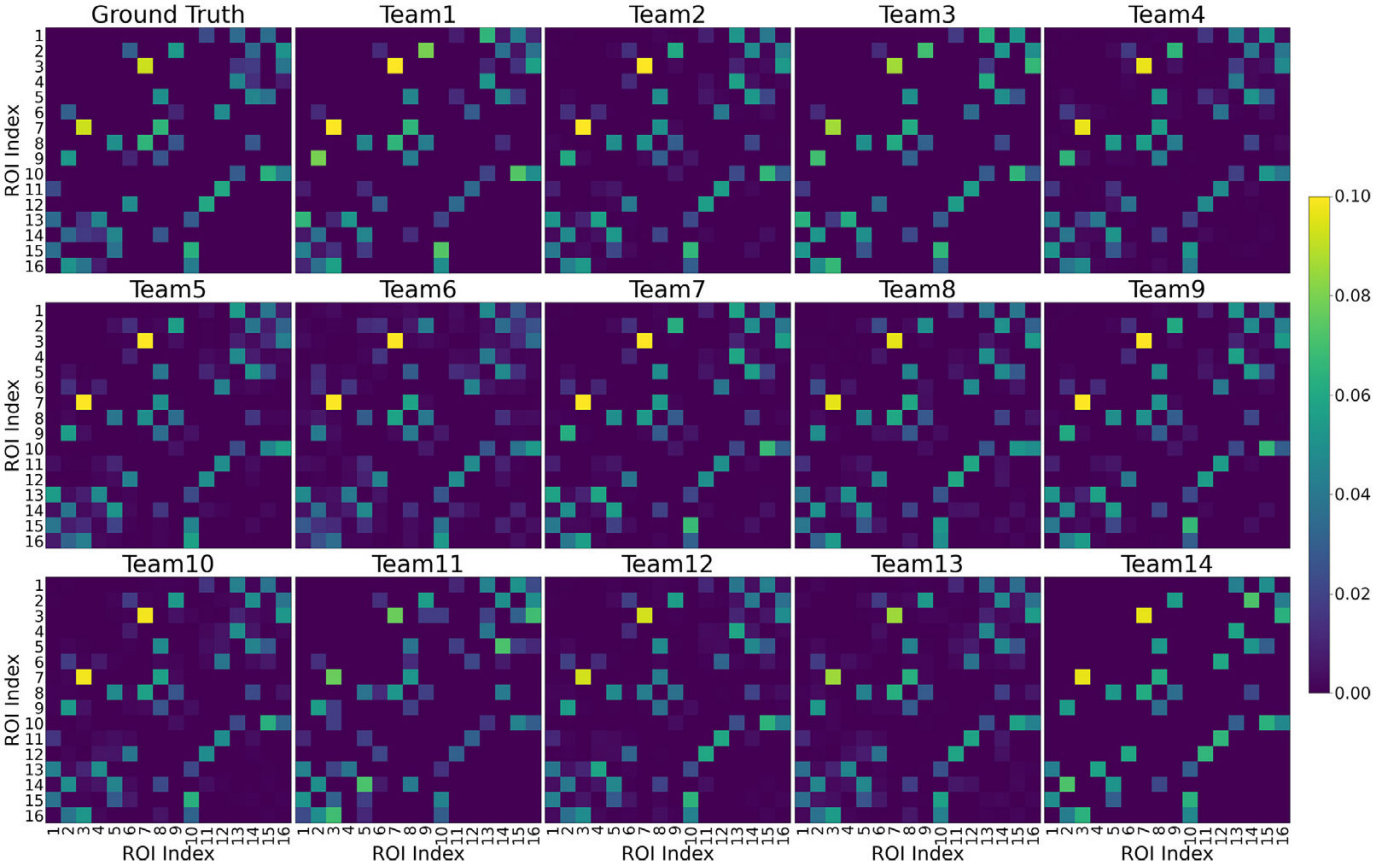
The test dataset’s ground-truth connectivity matrix (top left) and each team’s best-performing classification matrices. All matrices are symmetric, and the upper triangular matrices are normalized to sum to one. The 26 non-zero connections of the test dataset have weights ranging from 0.008 to 0.092.

Fourteen teams participated in the DiSCo challenge and submitted 111 connectivity matrices for the test dataset. Fig. 2A shows the Pearson correlation coefficient *r* between the participant’s submitted matrices and the ground-truth connectivity matrix of the validation dataset. Fig. 2B shows the fraction of valid connectivity weight in pairs of connected regions (non-zero connection strength) in the ground-truth connectivity matrix. The best-performing matrix of each team ranges from *r* = 0.874 to *r* = 0.973 (mean *r* = 0.950). The area under the ROC curve (AUC), computed from the submitted matrices and the ground-truth binary connectivity matrix, is reported in Fig. 2C. Fig. 2D shows the accuracy of all methods when thresholding the submitted matrices at 5% of their maximum value. The ground-truth connectivity matrix of the test dataset is shown in Fig. 3, alongside each team’s best-performing method (method with maximum *r*).

**Fig. 4.**
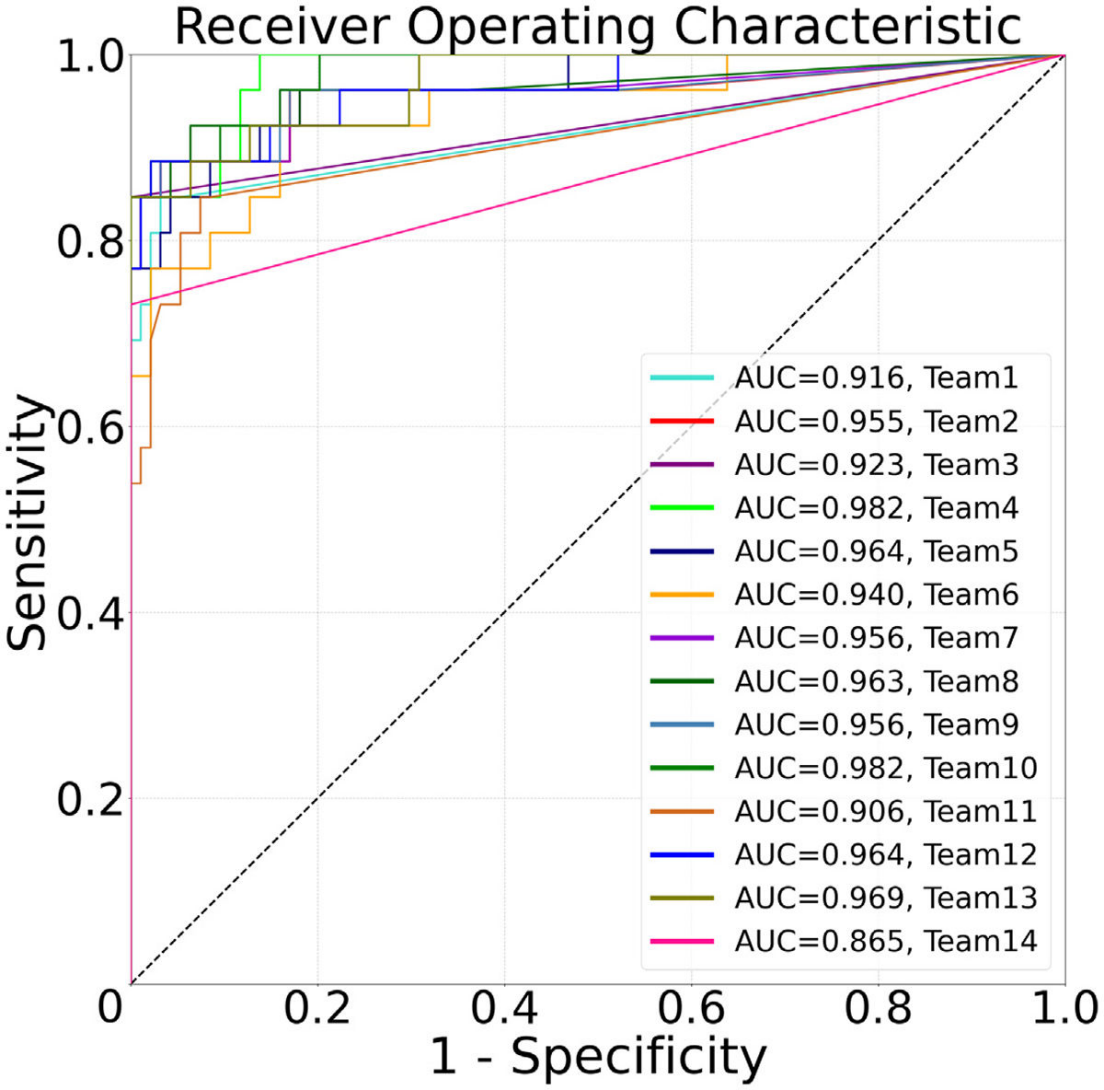
Receiver Operating Characteristic (ROC) curves of the submitted matrix with the highest correlation for each team. The black dashed line shows the performance of a connectivity matrix with randomly generated weights. The corresponding area under the curve (AUC) is reported in the bottom right panel.

**Fig. 5.**
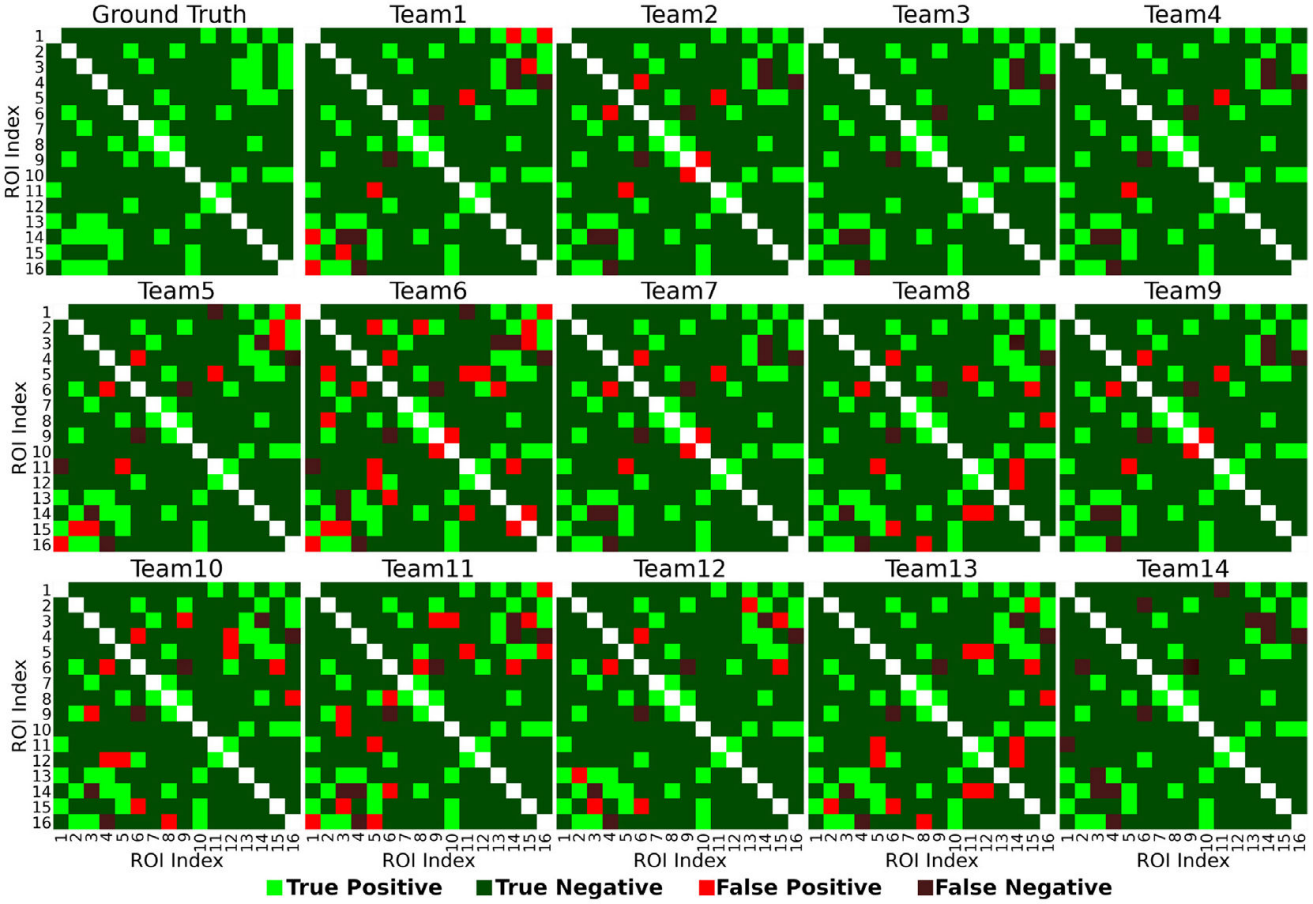
The test dataset’s ground-truth binary connectivity matrix (top left) and each team’s matrices. All matrices were thresholded at 5% of their maximal value. The light/dark green and light/dark red colours show the true positives/negatives and false positives/negatives, respectively. All matrices are symmetric.

Fig. 4 shows the ROC curves for the best-performing methods of each team. The corresponding area under the curve (AUC) is reported in the legend, ranging from 0.865 to 0.982 (mean AUC=0.946). Fig. 5 shows the ground-truth binary connectivity matrix (top left) and each team’s pairs of ROIs classifications. Matrices were thresholded at 5% of their maximal value. The light green and dark green colours show the true positives and true negatives, respectively. The light red and dark red colours show the false positives and the false negatives, respectively.

**Fig. 6.**
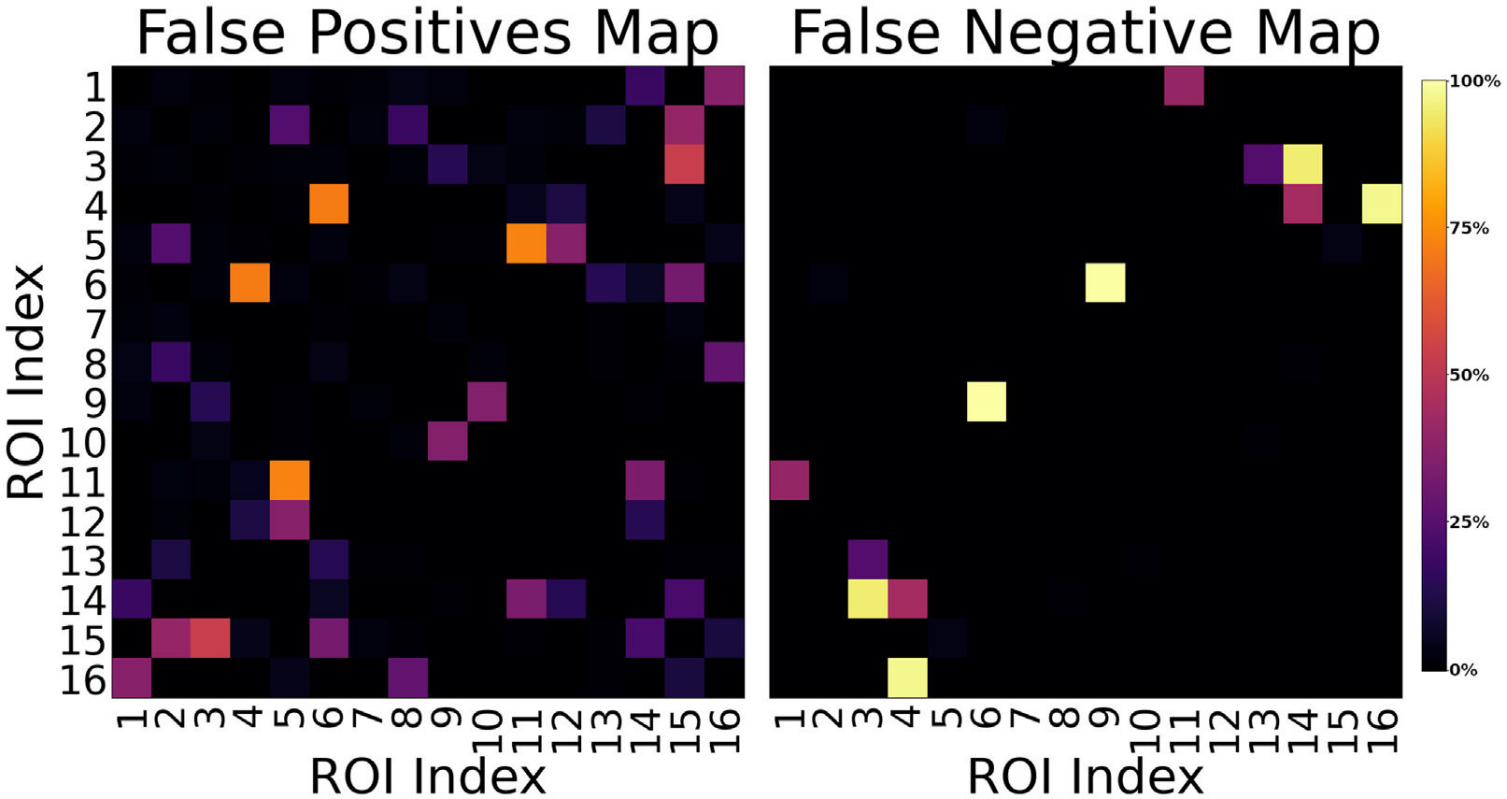
Percentage of classification error for each pair of ROIs for the submitted matrices (111) and using the threshold at 5% of their maximal value. The left subfigure reports the false positive connections. Regions 5–11 and 4–6 show the worst performance, with 73% (81) and 71% (79) matrices erroneously identifying them connected. The right subfigure reports the false negative connections. Regions 6–9, 4–16, and 3–14 show the worst classification, with 100% (111), 97% (108) and 95% (105) of methods erroneously identifying them as not connected. Both matrices are symmetric.

**Fig. 7.**
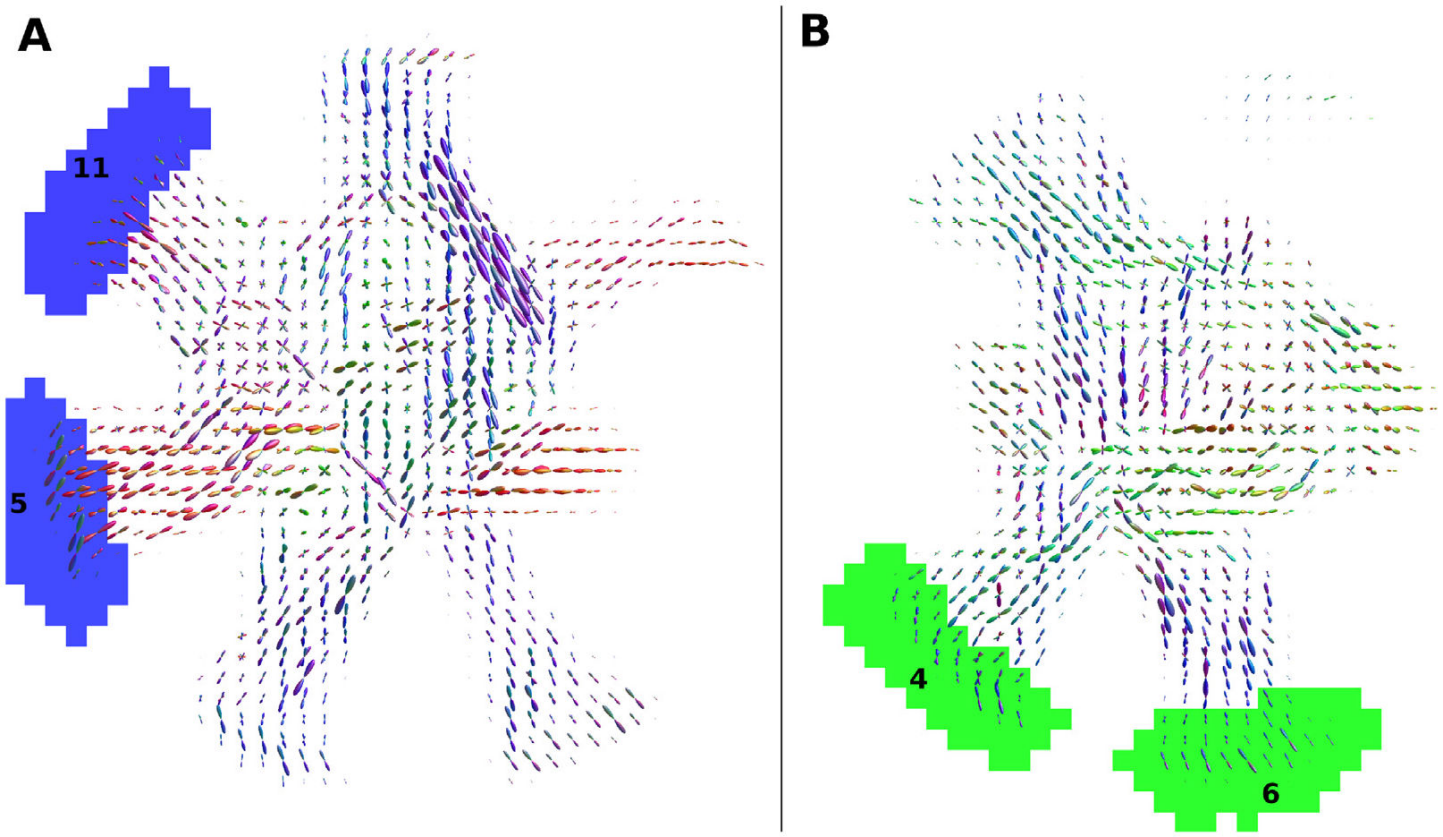
False positive bundles connecting ROIs 5–11 (A, blue) and 4–6 (B, green). These 2 pairs of regions have been incorrectly identified as connected by 73% and 71% of the submitted matrices, using a threshold at 5% of their maximal value, respectively. Glyphs show the local orientations of the ground-truth tubular fibres intersecting voxels, coloured with their orientation (left-right: red, anterior-posterior: green, superior-inferior: blue). Both pairs of regions are spatially located next to each other.

**Fig. 8.**
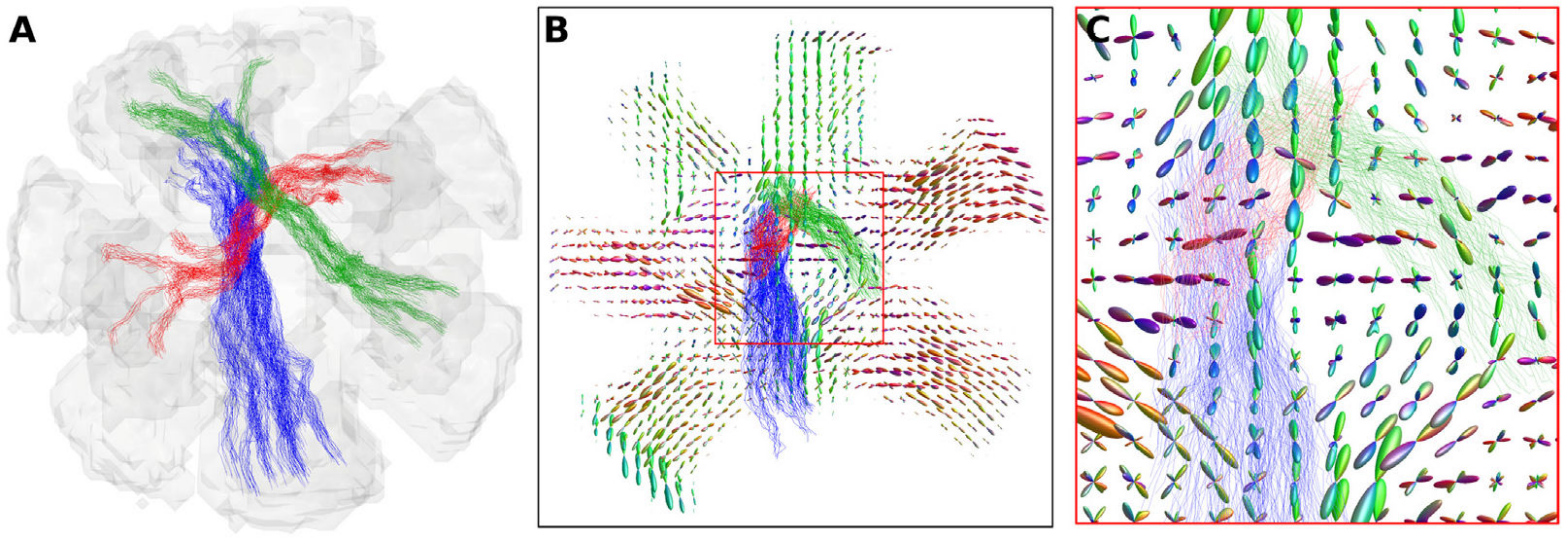
False negative bundles connecting ROIs 6–9 (green), 4–16 (red), and 3–14 (blue), were erroneously reported non-connected by 100%, 97% and 95% of methods, respectively. A) show a 3D rendering of the ground-truth fibre trajectories of the three bundles. B) and C) show a 2D cross-sectional image of the local orientations of the ground-truth tubular fibres, with fibre segment intersecting the 2D plane. All three bundles show a long and straight configuration going through the centre of the phantom and mixing with the other bundles. Those three bundles are the bundle with the lowest, second lowest and 5th lowest connectivity in the ground-truth weighted connectivity matrix.

The percentage of classification error for each pair of ROIs for all submitted connectivity matrices is shown in Fig. 6. The left subfigure reports the false positive connections. The worst performance is reported for ROIs 5–11 and 4–6 with 73% and 71% of matrices erroneously identifying them as connected. The right subfigure reports the false negative connections, with ROIs 6–9, 4–16, and 3–14 showing the worst classification, with 100%, 97% and 95% of methods erroneously identifying them as not connected, respectively, although connected in the ground-truth. Fig. 7 shows the location of the false positive bundles connecting ROIs 5–11 (blue) and 4–6 (green). Both pairs of ROIs are spatially located next to each other. Fibre ODFs show the corresponding ground-truth numerical fibre distribution. Fig. 8 shows the false negative bundles connecting ROIs 6–9 (green), 4–16 (red), and 3–14 (blue). They are the bundle with the lowest, second lowest and 5th lowest connectivity in the ground-truth weighted connectivity matrix. All three bundles show long and straight configurations going through the centre of the numerical phantom.

Each team’s best-performing method processing steps are listed in Table 1. All teams submissions are described in supplementary material.

## 4. Discussion

The aim of this work was to test tractography algorithms in carefully designed numerical phantoms with intricate connectivity patterns. The challenge was to identify connected pairs of ROIs among 16 ROIs and estimate their connection strength, defined as the cross-sectional area of the synthetic fibres interconnecting them. The DiSCo challenge phantoms were developed to feature challenging configurations found in the human brain, such as branching, crossing, and tortuous trajectories. Although these phantoms don’t mimic the anatomy of the human brain, they provide valuable data for studying tractography and connectivity. As such, results obtained on the DiSCo dataset are not directly transferable to real brain data. Rather, they should be used to evaluate the relative performance among connectivity methods. Unlike traditional tractography numerical phantoms that use biophysical models, the DiSCo datasets were obtained from realistic Monte Carlo simulations. This approach allows for a signal with rich microstructure and complex and coherent macrostructure properties, suitable to study properties of connectivity methods.

Participating teams did remarkably well, despite the known limitations of diffusion tractography methods (Jbabdi et al., 2015; Jones, 2010). This is shown by the large fraction of connection weight reported in the pair of ROIs connected in the ground-truth matrix (0.89 on average, see Fig. 2A). Methods generally showed high accuracy (average of 0.91) and high AUC (average of 0.95) for the identification of connected/non-connected ROIs (Fig. 2C,D). Overall, the mean Pearson’s correlation coefficient across all submissions is *r* = 0.95, with a maximum of *r* = 0.973 (see Fig. 2B). Despite the macroscopic complexity of the numerical phantom, state-of-the-art tractography methods combined with state-of-the-art spherical deconvolution methods can correctly identify connected ROIs, producing connectivity results predominantly faithful to the numerical substrate.

### 4.1. Correlation coefficients with the ground-truth weights

The correlation coefficients obtained on numerical data are higher than those reported in brain connectivity studies comparing DW-MRI weights estimation and labelled cell counts from tracing studies in the intraparietal sulcus (Caminiti et al., 2021) (*r* = 0. 65) and intrahemispheric (Donahue et al., 2016) (*r* = 0.59) connections. This highlights that the DiSCo numerical substrates oversimplifies the complexity of real MRI signals. Indeed, tractography limitations could originate from other factors aside from the diffusion information, such as MRI artifacts (B0 field inhomogeneity, susceptibility, motion, etc) and region-dependent T2 effects (Le Bihan et al., 2006). Despite the complexity achieved with the DiSCo numerical phantoms, real tissue shows a higher heterogeneity (Andersson et al., 2021) that was not reproduced, which may affect the relevance of some findings on biological tissue data. However, it is possible to know the ground-truth connectivity with higher accuracy than tracing studies, including the trajectory and diameter of the numerical fibres and the voxelwise compartmental volume fractions. Future studies should investigate the effects of MRI artifacts and signal-to-noise ratio on the connectivity estimation. New numerical datasets should be generated with varying numbers of ROIs, ROI sizes, and connectivity strenghts. This would allow testing DW-MRI connectivity estimation methods in diverse and complex environments, improving the generalizability of our results. In addition, other evaluation metrics, such as Dice similarity coefficient, could be used to test bundle volume identification using tractography. Moreover, research should be done on combining tractography performances in a single measurement as different measurements can lead to a change in the ranking of a specific method, sometime in opposite directions.

### 4.2. Binary classification of the connectivity

The performances of participating teams for binary classification of the connectivity is also higher on the DiSCo numerical phantoms than previously reported results on other synthetic data (Maier-Hein et al., 2017) and real brain (Caminiti et al., 2021; Donahue et al., 2016; Girard et al., 2020). For instance, teams 3 and 14 obtained a specificity of 1, i.e. no false positives (see Figs. 4, 5). Most teams have 3 to 5 false negatives, showing high sensitivity. Team 3 and 4 have the highest accuracy, with 4 and 5 misclassified pairs of ROIs, respectively, out of 120 pairs of ROIs. Moreover, Team 3 and 14’s best-performing methods had no false positives, even before applying the thresholding. This was achieved by the teams via thresholding of their matrices before the challenge submission, with a threshold value estimated using the training dataset. This also suggest the DiSCo substrates, although complex, are oversimplifying real brain connectivity.

Nonetheless, the errors (false positives/negatives) of methods are not randomly distributed among the connections of the numerical substrate. Rather, a subset of bundles is either consistently wrongly connected or wrongly not connected (see Fig. 6). The most frequently reported false negatives are non-dominant bundles with generally low connection strength in the ground-truth matrix (fewer synthetic fibres than other bundles). They also have a straight geometric profile with synthetic fibres crossing with several other bundles in the central partition of the phantom, as shown in Fig. 8. Contrarily, the most frequently reported false positives are bundles connecting adjacent ROIs (see Fig. 7). These bundles are likely the result of two portions of existing bundles wrongly merged due to a low angle crossing and bottlenecks configurations (Girard et al., 2020; Maier-Hein et al., 2017). This may indicate that bundle metrics, such as volume and structural connectivity estimates, may be biased by the shape and size of white matter bundles, rather than being uniform across all of them.

In this work, we fixed a threshold of 5% of each method maximum connectivity to binarise connectivity matrices. This will inevitably penalise the identification of connections with low weights, where under-estimation may lead to the exclusion of connections. This is also the case *in vivo*, when connectivity matrices are binarised. As such, alternative matrix binarisation methods, such as using various fixed thresholds or using thresholds specific to each connection, should be investigated in future work.

### 4.3. Characteristics of the best-performing methods

The estimated connectivity matrices of the best-performing methods submitted by the teams are shown in Fig. 3, and their corresponding processing methods are listed in Table 1. Most of the team used the MPPCA denoising algorithm (Veraart et al., 2016) before performing the local reconstructions. Although multiple local reconstruction methods (Baete et al., 2016; Canales-Rodríguez et al., 2015; Coronado-Leija et al., 2017; Sedlar et al., 2021; Tournier et al., 2004; Wu et al., 2019) yield a high Pearson correlation coefficient, the multi-shell multi-tissue spherical deconvolution method was the most common (Jeurissen et al., 2014). Various tractography algorithms were selected (Aydogan and Shi, 2021; Garyfallidis et al., 2014; Tournier et al., 2010; 2019; Wu et al., 2020; Yeh, 2017), with the probabilistic streamlines tractography methods being the most common. In particular, the top 3 connectivity methods with the highest Pearson correlation coefficient (*r*) all used the Parallel Transport Tractography (PTT) algorithm (Aydogan and Shi, 2021). Notably, the method with the highest accuracy used the RK4 deterministic tractography algorithm (Yeh, 2017) combined with the Radial DSI reconstruction (Baete et al., 2016). Moreover, most of the submitted matrices used microstructure-informed tractography (Daducci et al., 2014; Frigo et al., 2021; Smith et al., 2013; 2015) to weigh the connectivity matrices, in particular, the top 3 all used the SIFT2 (Smith et al., 2015) or the COMMIT (Daducci et al., 2014) methods. However, teams using streamline counts or thresholded streamline counts to estimate the connectivity also obtained a high Pearson correlation coefficient, particularly when paired with deterministic tractography algorithms. Future work should target evaluating individual steps (e.g. denoising, local re-construction, tractography, connectivity weighting methods), fixing the other steps to assess it effects on the connectivity evaluation. Moreover, other methods, not selected by teams, may provide similarly good results and shouldn’t discarded. Rather, results presented here serve as baseline for future method testing and development. Nonetheless, the geometry of the fibre in DiSCo substrates may favour some methods over others. Hence, conclusions derived from numerical substrates must be challenged against real data.

## 5. Conclusion

Current tractography and connectivity methods show exceptional performance on the DiSCo datasets. All methods selected by participating teams were able to accurately estimate connectivity weights corresponding to the cross-sectional area of the synthetic fibres connecting the network. Furthermore, they were able to accurately identify the pairs of ROIs interconnected by synthetic fibres. Previous phantoms were designed to validate either tractography or microstructure; we believe that DiSCo phantoms enable an improved assessment of the reliability of quantitative connectivity methods thanks to their microscopic and macroscopic properties. Tractography is capable of accurately solving complex configurations, as demonstrated by this challenge. However, a noticeable gap exists between the challenge results and results in real data or from other validation techniques. As such, the complexity of the numerical substrates should be improved, for instance, by varying the tubular shape of the fibre, increasing the packing density, adding T2 effects and simulating membrane permeability. Moreover, future work should modify the DW-MRI signal by adding MRI artifacts, changing spatial and angular resolutions, as well as varying the acquisition protocol to test tractography in clinically realistic DW-MRI signals. Overall, this work contributes to the growing body of evidence suggesting that tractography research should focus on improving tractography in bottlenecks and other challenging fibre configurations. The DiSCo datasets are available publicly (Rafael-Patino et al., 2021a; 2022) to foster the development of the next generation of structural connectivity methods.

## Supplementary Material

Supplementary Data S1

## Figures and Tables

**Table 1:** Best-performing method for each team. Most of the best-performing methods used DW-MRI signal denoising, multi-shell multi-tissue spherical deconvolution, probabilistic or deterministic tractography, and microstructure informed-tractography filtering approaches. ASI (Wu et al., 2019), AxCaliber (Assaf et al., 2008; Fick et al., 2019), COMMIT (Daducci et al., 2014), COMMIT2_tree_ (Ocampo-Pineda et al., 2021), CSD (Tournier et al., 2004; 2019), Deterministic RK4 (Yeh, 2017), iFOD2 (Tournier et al., 2010; 2019), iFOD1 (Tournier et al., 2010; 2019), MPPCA (Veraart et al., 2016), MRDS (Coronado-Leija et al., 2017), msmt-CSD (Jeurissen et al., 2014), ms-fODF (Tran and Shi, 2015), Probabilistic tractography (Garyfallidis et al., 2014), RUMBA-SD (Canales-Rodríguez et al., 2015), Radial DSI (Baete et al., 2016), SD_STREAM (Tournier et al., 2019), SIFT2 (Smith et al., 2015), SR-ASI (Wu et al., 2020), PFT (Girard et al., 2014), PTT (Aydogan and Shi, 2021), U-net fODFs (Sedlar et al., 2021).

	*r*	Fraction of Valid Streamlines	AUC	Accuracy	Denoising	Local Modelling	Tractography Algorithm	Connectivity Weighting
Team 1	0.945	0.966	0.918	0.942	MPPCA	RUMBA-SD	Probabilistic	counts
Team 2	0.960	0.937	0.955	0.942	MPPCA	msmt-CSD	SD_STREAM	SIFT2
Team 3	0.951	**1.000**	0.923	**0.967**	MPPCA	Radial DSI	Deterministic RK4	count, length scaling,thresholding
Team 4	0.964	0.930	**0.982**	0.958		U-net fODFs	iFOD2	SIFT2
Team 5	0.940	0.900	0.964	0.925		msmt-CSD	iFOD2	SIFT2
Team 6	0.919	0.856	0.940	0.858	MPPCA	ASI	SR-ASI	SIFT2
Team 7	0.954	0.938	0.956	0.942	MPPCA	msmt-CSD	SD_STREAM	counts
Team 8	0.971	0.930	0.963	0.925	MPPCA	msmt-CSD	PTT	COMMIT2_tree_
Team 9	0.960	0.938	0.956	0.942	MPPCA	msmt-CSD	SD_STREAM	SIFT2
Team 10	0.972	0.911	**0.982**	0.925	MPPCA	msmt-CSD	PTT	SIFT2
Team 11	0.874	0.861	0.906	0.900		CSD	PFT	COMMIT
Team 12	0.964	0.912	0.964	0.942	MPPCA	msmt-CSD	iFOD1	AxCaliber
Team 13	**0.973**	0.893	0.969	0.917	MPPCA	ms-fODFs	PTT	COMMIT
Team 14	0.946	**1.000**	0.865	0.942	MPPCA	MRDS	iFOD2	count, thresholding

## Data Availability

The three dataset used in this study is available online at http://data.mendeley.com/datasets/fgf86jdfg6 (Rafael-Patino et al., 2021a; 2022). The dataset includes the noiseless and corrupted DW-MRI signal (SNR = [10, 20, 30, 40, 50]) at two resolutions (25 *μm* and 50 *μm* isotropic voxels). The dataset also includes the 3D mesh used to simulate the signals, the ROIs masks, and the trajectories of the fibres with their corresponding diameter.
