# Supplementary material for: Tractography passes the test: Results from the diffusion-simulated connectivity (disco) challenge
Source: Neuroimage. Author manuscript; Available in PMC 2024 Jan 6. (PMC10771037; doi:10.1016/j.neuroimage.2023.120231)
Supplement: Supplementary Data S1 [file NIHMS1950882-supplement-Supplementary_Data_S1.pdf]

Tractography passes the test: results from the diffusion-simulated connectivity (DiSCo) challenge

---

Supplementary material

Team 1

Our submission pipelines were evaluated on different subsets DW-MRI measurements: submissions 1-3 with b=[13,191] s/mm^2, submissions 4-6 with b=[3,094; 13,191] s/mm^2, submissions 7-9 with b=[1,925, 3,094, 13,191] s/mm^2, and submission 10 with b= [1,000, 1,925, 3,094, 13,191] s/mm^2. A white matter mask for each dataset was found by fitting a 2-class Hidden Markov Random Field tissue classifier (Garyfallidis et al., 2014; Zhang et al., 2001) on an Anisotropic Power Map (Dell'Acqua et al., 2013). This map was extracted using a Q-Ball model fit with a spherical harmonic order of 8 on the 13,191 s/mm^2 b-shell. Some submissions (1, 2, 4, 5) used a denoised version of the data using Marchenko-Pastur Principal Component Analysis (MPPCA) with a patch radius of 4 (Manjón et al., 2013; Garyfallidis et al., 2014). All other submissions used no denoising. Fibre orientation distribution functions (ODFs) were estimated using the RUMBA-SD method (Canales-Rodríguez et al., 2015) implemented in DIPY (Garyfallidis et al., 2014) using various single-shell and multi-shell response functions. Moreover, some submissions were made using the total variation spatial regularization of RUMBA-SD. The number of iteration used with RUMBA-SD was also changed on submissions.

Probabilistic tractography was employed for all submissions (Garyfallidis et al., 2014). The tracking was conducted with a max angle of 20 degrees, a fibre ODF threshold of 0.1, a step size of 0.2, and using 27 seeds per voxel of the ROIs. A binary stopping criterion was used with the estimated white matter mask. All other parameters were left to their default settings. Outlier streamlines were filtered out by applying a Cluster Confidence Index (CCI) threshold (50) to each bundle connecting an ROI pair (Garyfallidis et al., 2014; Jordan et al., 2018). The connectivity matrix reflects the streamline

counts between any ROI pair. The matrix was further processed by applying various sparsity thresholds (110-300) on the streamline counts.

Team 2

DW-MRI date were denoised using the MPPCA algorithm implemented in MRtrix3 (Coronado-Leija et al., 2017; Tournier et al., 2019; Veraart et al., 2016). The fibre ODFs were obtained using the multi-shell multi-tissue constrained spherical deconvolution (MSMT-CST) algorithm (Jeurissen et al., 2014; Tournier et al., 2019).

Tractography was performed using deterministic algorithm (SD_STREAM; submissions 1-6) and probabilistic algorithm (iFOD2; submission 7-10), producing 1 million streamlines. SIFT (Smith et al., 2013; Tournier et al., 2019) was applied to filter tractograms to 100,000 streamlines (submission, 1-4, 9, 10) and SIFT2 (Smith et al., 2015; Tournier et al., 2019) to obtained streamline weighting (submissions 5-8). All connectomes were weighted using the filtered tractograms. Matrices of submission 2, 3, 8 and 10 were thresholded at 10% of their maximal value (Tournier et al., 2019). Weights of the matrices of submission 3, 4, 8, 9, 10 were scale by the bundle length (Tournier et al., 2019).

Team 3

The input DW-MRI images were denoised using the MPPCA algorithm implemented in MRtrix3 (Coronado-Leija et al., 2017; Tournier et al., 2019; Veraart et al., 2016), with the default parameters. We computed ODFs using the Radial DSI reconstruction (Baete et al., 2016) with the sampling length parameter edge=1.3. For this, we used the MATLAB implementation of the method available at: *bitbucket.org/sbaete/rdsi_recon*. Next, we ran our in-house MATLAB implementation of ODF-Fingerprinting (Baete et al., 2019) available at: *bitbucket.org/sbaete/odffingerprinting*. To this end, we generated a randomized ODF-dictionary of 1,000,000 elements (Filipiak et al., 2021) using the multicompartment diffusion model (Assaf et al., 2004), with $0 \leq N \leq 3$ fibres per voxel. In our ODF-dictionary, the compartment volumes of free water $p_{iso} \geq 0$ and neurites $p^{(i)} \geq 0$ summed up to 1. The intra-axonal fraction sizes were $f_{in} \in [0, 1]$ and the ranges of diffusivities were chosen as follows: free water $D_{iso} \in [2, 3] \times 10^{-9} m^2/s$, intra-axonal $D_{a,\|} \in [1.5, 2.5] \times 10^{-9} m^2/s$, extra-axonal $D_{e,\|} \in [1.5, 2.5] \times 10^{-9} m^2/s$, $D_{e,\perp} \in [0.5, 1.5] \times 10^{-9} m^2/s$. The b-values matched the input data, i.e. b = 1000, 1925, 3094, 13191 s/mm^2. For obtaining ODF-fingerprints from the

simulated data, we used Radial DSI reconstruction with the sampling length parameter edge=1.3.

We ran the RK4 deterministic tractography algorithm implemented in DSI Studio (Yeh, 2017). A seeding region was placed in the whole phantom. The quantitative anisotropy (QA) threshold was 0.205553. The angular threshold was 60 degrees. The step size was randomly selected from 0.5 voxel to 1.5 voxels. The fibre trajectories were smoothed by averaging the propagation direction with 20% of the previous direction. Tracks with length shorter than 4 or longer than 60 voxels were discarded. A total of 100,000 seeds were placed. The submitted connectivity matrices were computed in DSI Studio (Yeh, 2017). The connectivity matrix of submission 1 was obtained from counts of streamlines connecting ROIs thresholded at 10% of the sum. Submission 2 additionally filtered out the value below 1000. For submission 3, counts of streamlines divided by their median lengths, then thresholded at 10% of the sum. Submission 4 was done similarly, with further removing all connection below 20.

Team 4

The white matter (WM) mask was obtained by selecting the voxels with $FA \geq 0.35$ and dilating the obtained mask two times (one voxel each time). The grey matter (GM) mask was computed as the binarization of the given ROIs. Voxels where GM and WM overlapped were assigned to GM. The cerebrospinal fluid (CSF) mask was obtained as the complement of the union of the WM and GM masks. Submissions 1, 2, 5-8 used msmt-CSD performed with MRtrix3 (Dhollander et al., 2016; Jeurissen et al., 2014; Tournier et al., 2019). Submissions 3 and 4 used an in-house method based on spherical U-nets (Sedlar et al., 2021). We trained the model on synthetic data generated using the Dmipy toolbox (Fick et al., 2019). We used a multi-compartment model with three compartments: ball – isotropic Gaussian for cerebrospinal fluid signal modelling, zeppelin – anisotropic Gaussian for extra-axonal signal modelling and gamma distributed sticks for intra-axonal signal modelling. Isotropic, intra- and extra-axonal parallel diffusivities are drawn from uniform distribution $U[0.54, 0.66] \times 10^{-9} m^2/s$, extra-axonal perpendicular diffusivity is defined using tortuosity model (Szafer et al., 1995). Isotropic and intra-axonal volume fractions are drawn from uniform distributions $U[0, 0.25]$ and $U[min(v_{IC}^{ph}) - 0.1 \times mean(v_{IC}^{ph}), max(v_{IC}^{ph}) + 0.1 \times mean(v_{IC}^{ph})]$ where v_{IC}^{ph} contains the ground truth intra-tubular volume fractions. Axon diameters d_a are drawn from uniform distribution

$U[min([d_a^{ph} - 0.1 \times mean(d_a^{ph}), max([d_a^{ph} + 0.1 \times mean(d_a^{ph})]$, where d_a^{ph} contains ground truth strand diameters. Shape parameter of gamma distribution κ is drawn from uniform distribution $U[0.45, 0.55]$ and scale parameter $\theta = 0.5 \times \frac{d_a}{\kappa}$. We have generated in total 50,000 single fibre samples with orientation $(0, 0)$. The fibre ODFs were estimated using the spherical U-net model adjusted from Sedlar et al. (2021). It contains 4 contracting and four expanding layers, with in total 12,929 trainable parameters. The model is trained by minimizing mean squared error between ground truth and estimated fibre ODFs over 3000 iterations with Adam optimizer with learning rate 0.001. In each iteration, 128 dMRI synthetic samples are generated with 1, 2 or 3 fibre bundles with random orientations, such that the crossing angles between the two bundles are at least 20°. Samples were corrupted with Rician noise (SNR=30).

Probabilistic tractography was performed with the iFOD2 algorithm (Tournier et al., 2010, 2019) and Anatomically-Constrained Tractography (Smith et al., 2012). Tractography was initiated from the GM-WM interface to generate 1 million streamlines. Submissions 1 and 3 used tractography filtering performed with the Mrtrix3 implementation of SIFT2 (Smith et al., 2015; Tournier et al., 2019). Submissions 2 and 4-8 used tractography filtering performed with TALON (Frigo et al., 2021) with the intra-cellular volume fraction map computed with Dmipy (Fick et al., 2019) from a spherical mean multi-compartment model with 2 watson-distributed bundles (stick&zeppelin, $\lambda_{\|} = 1.7 \times 10^{-9} m^2/s$, tortuous $\lambda_{\perp}$) and one isotropic compartment (ball with $\lambda_r = 3 \times 10^{-9} m^2/s$). A group sparsity regularization term was additionally employed for submission 5-8. Groups of streamlines were defined by clustering streamlines according to the pairs of ROIs they connected. The regularization parameter σ of submission 5-8 was set as 0.0001, 0.0004, 0.0021, 0.01, respectively. Each streamline was associated to two ROIs according to a radial search around its endpoints with a radius of 2 mm (Tournier et al., 2019). Each entry of the connectivity matrices were defined as the sum of the streamline weights (SIFT2 and TALON) for the streamlines connecting the two ROIs.

Team 5

MRtrix3 software (Tournier et al., 2019) was used to perform the analyses. An unsupervised method was used to estimate the WM, GM, and CSF tissue response functions (Dhollander et al., 2016). The fibre ODF in each voxel was estimated using msmt-CSD (Jeurissen et al., 2014; Tournier et al., 2019).

Probabilistic tractography was performed using iFOD2 (Tournier et al., 2010, 2019). Streamlines were seeded from a binary mask formed from the intersection of white-matter voxels (indicated by a white-matter response function greater than 0.1) and a dilated mask of the 16 ROIs. One hundred thousand streamlines were estimated in total. SIFT2 (Smith et al., 2015; Tournier et al., 2019) was used to determine the cross-sectional area multiplier for each estimated streamline. These values were later used to created connectivity matrices in which each streamline's contribution to connectivity was scaled by its cross-sectional area multiplier to reflect streamline densities. Connectivity matrix were constructed from streamline counts generated by the probabilistic CSD tractography (submissions 1, 3) or ii) the weighted streamline counts using SIFT2 streamline multipliers (submissions 2, 4-10). Element e of these connectivity matrices were log-transformed following $f(e) = log(1 + e)$ (submission 3-10). After, the connectivity matrices were thresholded with a threshold ϵ to replace small connectivity weights (which are most potentially due to noise/randomness in probabilistic CSD) with zeros (submissions 3-10). Finally, a shifting operator function (provided below) were used to reduce the gap created by thresholding (submissions 5-10). The shifting is controlled by a parameter α which shifts all non-zero edge weights as a function of minimum non-zero connectivity weight (e_{min}) $g(e) = e - \alpha e_{min}$. These parameters were tuned on the training data and validated using the validation dataset. The optimal threshold (ϵ) was found to be $log(600)$. The shifting scale (α) were set to 0.4, 0.6, 0.65, 0.7, 0.75, 0.8, for submissions 5-10, respectively.

Team 6

DW-MRI data were denoised using Marchenko-Pastur PCA (Cordero-Grande et al., 2019; Tournier et al., 2019). Gibbs ringing artifacts were removed using the local subvoxel-shifts (Kellner et al., 2016; Tournier et al., 2019). B1 field inhomogeneity correction was performed using the N4 algorithm available in ANTs software package (Tustison et al., 2010). Diffusion gradient orientation correction (Wu et al., 2020f), DW-MRI data upsampling (Wu et al., 2020b, 2021a) to 0.5 mm isotropic voxel size, and phantom mask estimation (Wu et al., 2020a), were also performed. Fibre ODFs were estimated using the Asymmetry Spectrum Imaging (ASI) method (Wu et al., 2019, 2020e,c). ASI fits a mixture of asymmetric fibre ODF to the diffusion signal. We employed the super-resolution asymmetry spectrum imaging to perform tractography, which characterizes high-resolution intravoxel ar-

chitecture structure and microstructure tissue components. ASI represented the diffusion signal as a multi-level intravoxel architecture structure and harnessed a biologically-motivated constraint from the spatially-continuous nature of fibre tracts.

Tractography is generated using Super-Resolution Asymmetry Spectrum Imaging (SR-ASI) algorithm (Wu et al., 2019, 2020e, 2021b). Tractograms are generated by successively following local directions determined from the asymmetric fibre ODFs. We adaptively update the forward direction via a scouting mechanism based on the asymmetric fibre ODF, and then the next position is updated with the adaptive step size. An automated unsupervised fibre clustering method was first applied to generate the fibre cluster (Wu et al., 2020d, 2021a). The streamlines were mapped to a Hilbert space by parameterizing as coefficients of cosine series, and then grouped into 200 clusters via K-medoids clustering. Streamlines with distances greater than 1.5 standard deviations from their cluster centres were removed as outliers, and finally, the remaining streamlines were combined as a single collection. We first employed the SIFT2 (Smith et al., 2015; Tournier et al., 2019) to optimize per-streamline cross-section multipliers to match the tractogram to fixel-wise fibre densities and estimate the final value of SIFT proportionality coefficient μ, without filtering the streamline. With the estimated μ, we then employed the SIFT (Smith et al., 2013) to filter the tractogram such that the streamline densities matched the FOD lobe integrals. Finally, we further filtered the tractogram using the COMMIT2 method Schiavi et al. (2020). We then employed SIFT2 to estimate a weighting factor for each streamline. The connectivity matrices were computed from the SIFT2 weights of the streamlines connecting two ROIs.

Team 7

The input DW-MRI images were denoised (Veraart et al., 2016) and corrected for Gibbs-ringing artifacts (Kellner et al., 2016) using MRTrix3 (Tournier et al., 2019). We created a binary mask of the brain using MRtrix3 (Tournier et al., 2019) to be used in future steps. We estimate response functions for three tissue types using Dhollander algorithm (Dhollander et al., 2016). We perform fibre ODF estimations to find the orientation of fibres in each voxel using msmt-CSD (Jeurissen et al., 2014; Tournier et al., 2019). Finally, we performed intensity normalization to correct for global intensity differences (Tournier et al., 2019).

We used deterministic tractography (SD_STREAM) with dynamic seeding to make 10,000,000 streamlines (Tournier et al., 2019). We removed extraneous streamlines using the SIFT (Smith et al., 2013; Tournier et al., 2019). For submissions 1 and 2, we kept 1,000,000 streamlines from the SIFT processing. For submissions 3 and 4, we let SIFT run until done and kept 250,000 streamlines. The connectivity matrices 1 and 3 were obtained from streamline counts. The connectivity matrices of submissions 2 and 4 were weighted by the cross-sectional area using SIFT2 (Smith et al., 2015; Tournier et al., 2019).

Team 8

Denoising was performed with MRtrix3 (Veraart et al., 2016; Tournier et al., 2019). We computed the intra-neurite volume fraction (INTRA) map using the Spherical Mean Technique (SMT) (Kaden et al., 2016). We used this map to create the WM mask by binarizing it, using as threshold the 82nd percentile.

The fibre ODF were estimated using the msmt-CSD algorithm (Jeurissen et al., 2014; Tournier et al., 2019) using the response function computed with Dhollander algorithm (Dhollander et al., 2016; Tournier et al., 2019). Then, we normalized the white matter fibre ODFs (Raffelt et al., 2017). We generated 1 million streamlines using Trekker (Aydogan and Shi, 2021) with default parameters, seeding from the WM mask and discarding streamlines shorter than 4 mm.

For submission 1, the tractogram was processed using the COMMIT (Daducci et al., 2014). We employed the ball&sticks model (Behrens et al., 2003), setting the parallel diffusivity to $0.6 \times 10^{-3} mm^2/s$ and using two isotropic diffusivities ($0.6 \times 10^{-3} mm^2/s$ and $1.0 \times 10^{-3} mm^2/s$) to model partial volume with CSF and GM. These diffusivities were defined using the intrinsic diffusivity (DIFF) map extracted with SMT (Kaden et al., 2016). To improve the fit with COMMIT in voxels with poor streamline coverage (Sairanen et al., 2021), we created a confidence map by applying a Gaussian smoothing to the WM mask boundaries.

For submissions 2-5, the resulting tractogram was processed using COMMIT2 (Schiavi et al., 2020) with the same forward model employed in submission 1. As regularization parameter for minimizing the number of bundles, we used $\lambda = 1 \times 10^{-4}$ for connectomes 2 and 3, and $lambda = 1 \times 10^{-3}$ for submission 4 and 5. To reduce possible biases from the regularization term of COMMIT2, we applied a final debiasing step to correct the magnitude of

the recovered coefficients in submissions 2 and 4 (Zou, 2006). For submission 6-9, the resulting tractogram was processed using COMMIT2$_{tree}$ (Ocampo-Pineda et al., 2021) with the same forward model employed in submission 1. The QuickBundles (Garyfallidis et al., 2012, 2014) threshold was set to 2 for submission 6 and 7, and to 4 for submissions 8, 9. The regularization parameter for minimizing the number of bundles was set to $\lambda = 2.5x10^{-4}$, $\lambda = 1.0x10^{-4}$, $\lambda = 7.5x10^{-3}$ and $\lambda = 1.0x10^{-4}$, respectively for submission 6-9. To reduce possible biases from the regularization term of COMMIT2$_{tree}$, we applied a final debiasing step to correct the magnitude of the recovered coefficients in submission 6 and 8 (Zou, 2006). Finally, for submission 10, the resulting tractogram was optimized using the Bundle-o-graphy method (Battocchio et al., 2020), which exploits a global optimization approach to adapt the bundle geometry to the observed DW-MRI data.

The connectivity matrices were constructed by summing the weights estimated for each streamline by the corresponding COMMIT weight.

Team 9

Image denoising has been performed as the first step of the image-processing pipeline. DW-MRI raw data have undergone noise level estimation and denoising based on random matrix theory (Tournier et al., 2019; Veraart et al., 2016). In this step we tested two different situations: DW-MRI denoising (submissions 1, 2, 3, 7, 9) vs no denoising (submissions 4, 5, 6, 8, 10).

Constrained Spherical Deconvolution (CSD) signal modelling has been employed in this step (Tournier et al., 2007, 2019). Response function estimation has been performed using the Dhollander algorithm (Dhollander et al., 2016; Tournier et al., 2019), which allows multi-shell, multi-tissue response function estimation without explicitly delineating a tissue segmentation.

Two different trajectory estimation methods have been evaluated: the SD_STREAM deterministic algorithm (submissions 1, 2, 4, 5, 9, 10) and the iFOD2 probabilistic (submissions 3, 6, 7, 8) (Tournier et al., 2019). In both cases, tractography has been performed with default tracking parameters by generating 1 million streamlines within the mask generated from the DW-MRI image.

Three different streamlines filtering methods (Smith et al., 2013, 2015) have been tested: SIFT (submissions 1, 5, 7), SIFT2 (submissions 2, 4, 6, 8) and no filtering (submission 3, 6, 9, 10). For the SIFT method, a total of 100,000 streamlines has been selected from the original 1 million streamlines.

Matrix weight estimation has been performed in different ways according to the post-processing strategy. For the unfiltered streamline approach, we used the number of streamlines between pairs of ROI (submissions 1, 5) and by scaling each contribution to the connectome edge by the inverse of the streamline length (submissions 9, 10). For SIFT, we computed the number of streamlines between pairs of ROIs. For SIFT2, the sum of streamline weights for each streamline connecting each pair of ROIs was used.

Team 10

Noise, Gibbs ringing and Rician bias were reduced using tools implemented in MRtrix3 (Cordero-Grande et al., 2019; Kellner et al., 2016; Veraart et al., 2016; Tournier et al., 2019). Fibre ODFs were estimated using constrained spherical deconvolution with multi-shell multi-tissue response function (Dhollander et al., 2016; Jeurissen et al., 2014; Tournier et al., 2019) using tools implemented in MRtrix3. This step is common for the submissions.

The seed mask for the streamlines was computed by thresholding (0.02) the constant term of the spherical harmonic representation of the fibre ODFs, and keeping the largest connected component, and subtracting the node regions. This step is common for the full set of submissions. Streamlines were generated using TREKKER (Aydogan and Shi, 2021) with bidirectional pathway entry constraints from the node regions. Two sets of streamlines were generated: 1 million streamlines (submission 1) and 10 million streamlines (submission 02).

Since the ground truth was referred to as the cross-sectional area, streamlines were assigned weights using SIFT2 (Smith et al., 2015; Tournier et al., 2019). Default parameters were used for submissions 1 and 2. Additional parameter combinations were attempted for additional submissions. We would like to note that the additional parameters combinations were not significantly different from the default combination for the training and validation datasets.

SIFT2 weights of the streamlines connecting each pair of the regions were summed to obtain the connectivity weights (Tournier et al., 2019). Default parameters were used for submissions 1 and 2. Additional parameter combinations were attempted for additional submissions. We would like to note that the additional parameters combinations were not significantly different from the default combination for the training and validation datasets.

Team 11

No denoising was performed on the input DW-MRI data.

Fibre ODFs (spherical harmonics order 8) were estimated using DIPY (Garyfallidis et al., 2014) on the b1000 shell data.

Tractography was performed using deterministic and probabilistic tracking implemented in DIPY (Garyfallidis et al., 2014) and the Particle Filtering Tractography (PFT) algorithm (Girard et al., 2014), seeding from both the WM mask and the WM-GM interface mask. Streamlines were post-processed using COMMIT (Daducci et al., 2014) with default parameters.

Connectivity was computed using DIPY (Garyfallidis et al., 2014). Connectivity was estimated using the COMMIT weights.

Team 12

The DW-MRI data were filtered using the Marchenko-Pastur PCA method (Tournier et al., 2019; Veraart et al., 2016)) for each b-shell separately, while the non-diffusion-weighted data were averaged across the replicas. This operation implicated the changes in the order of gradient directions and the size of the data handled in the next step of data processing.

Spherical deconvolution with three different response functions was employed (Tax et al., 2014; Tournier et al., 2004, 2013). The fibre ODFs were estimated using the non-negativity constrained super-resolved spherical deconvolution (Tournier et al., 2007, 2019).

To generate the tractograms two probabilistic algorithms were used: iFOD1 (submissions 1, 2, 4-10) and iFOD2 (submission 3) (Tournier et al., 2010, 2019). All tractograms were generated with minimal streamline length of 5 mm. The maximum angle range from 15° to 30° and the step size from 0.05 to 0.5 mm.

Finally, to filter the tractograms, the SIFT (Smith et al., 2013) was used. We use the AxCaliber methodology (Assaf et al., 2008) provided with the Dmipy Toolbox (Fick et al., 2019) to estimate the fibre diameters. Once we calculated the diameters for all voxels, we mapped the estimated values to each streamline from the post-processing stage. The cross-sectional area of a single fibre was taken then as the average calculated through the interpolated values over the streamline. Finally, we summed all the averaged cross-sectional areas between every two regions of interest to obtain the matrix weights.

Team 13

Images were denoised using MPPCA algorithm (Tournier et al., 2019; Veraart et al., 2016) with default parameters. Denoised images were resized to have an isotropic voxel dimension of 0.5 mm using MRtrix. Diffusion tensor imaging was performed for the whole image using DIPY (Garyfallidis et al., 2014), with default parameters. The initial masks were computed by thresholding the mean diffusivity value at 0.000387. Final masks are computed by iteratively dilating the initial masks 5 times (Tournier et al., 2019). The same preprocessing steps were used for all the submissions.

The compartment modelling approach proposed by Tran and Shi (2015) was used. This approach uses fixed diffusivity values for intra- (stick) and inter- (ball) axonal compartments. These values are set to be $0.3e^{-3}mm^2/s$ and $0.1e^{-3}mm^2/s$, respectively. Fibre ODFs are then represented with 16th order spherical harmonic functions. The same local modelling was used in all the submissions.

The Trekker software (dmritrekker.github.io) running parallel transport tractography (PTT) algorithm (Aydogan and Shi, 2021) was used for fibre tracking. The computed image mask is used as seed. For submissions 1, 2, 3, we used the following parameters: -seed_count 100000 -useBestAtInit -initMaxEstTrials 1000 -probeLength 0.125 -probeQuality 6 -minFODamp 0.01 -dataSupportExponent 0.5 -probeRadius 0.125 -probeCount 6. For submissions 4, 5, 6, we used the following parameters: -seed_count 100000 -useBestAtInit -initMaxEstTrials 1000 -probeLength 0.125 -probeQuality 6 -minFODamp 0.01 -dataSupportExponent 0.5.

Tractography post-processing was done using the COMMIT (Daducci et al., 2014) and SIFT2 (Smith et al., 2015) frameworks. SIFT2 is used with default parameters in submissions 3 and 6. COMMIT is used in the other submissions. For COMMIT, up to 4 peaks in fibre ODFs were used. Then a StickZeppelinBall model is used to assign weights on the streamlines with following parameters: For submissions 1 and 4: $d_{\|} = 0.3e^{-3}$, $d_{\perp} = []$, $d_{isos} = [0.1 \times 10^{-3}]$. For submissions 2 and 5: $d_{\|} = 0.6e^{-3}$, $d_{\perp} = [0.3 \times 10^{-3}]$, $d_isos = [0.6 \times 10^{-3} 0.3 \times 10^{-3}]$.

MRtrix3 was used to obtain the connectivity matrices using the SIFT2 and COMMIT weights.

Team 14

DW-MRI data were denoised and debiased using MRtrix3 (Gudbjartsson and Patz, 1995; Tournier et al., 2019; Veraart et al., 2016).

Local orientations were obtained using the MRDS method (Coronado-Leija et al., 2017) to compute at most 4 diffusion compartments per voxel.

Tractography was performed using the iFOD2 algorithm (Tournier et al., 2019). All parameters were set to their default value, except *-cutoff* which was set to 0.05 (Tournier et al., 2010). 1,000,000 streamlines were generated with seeds randomly placed in the WM mask.

Streamlines not connecting two ROIs were removed. For submission 1, the computation of the connectivity matrix based on streamline counting. Values inferior to $0.16 \times max(M)$, were set to 0. This value was tuned on the training dataset. For submission 2, streamlines were weighted using COMMIT (Daducci et al., 2014) with the cylinder, zeppelin & ball model, with parameters diameters = [1.5, 2.0, 2.5, 3.0, 3.5, 4.0, 5.0, 6.0]μm, $d_{||} = 0.6 \times 10^{-9} m^2/s$, $d_{\perp} = 0.15 \times 10^{-9} m^2/s$, $d_{isos} = [0.35, 0.6] \times 10^{-9} m^2/s$.

References

Assaf, Y., Blumenfeld-Katzir, T., Yovel, Y., Basser, P.J., 2008. Axcaliber: a method for measuring axon diameter distribution from diffusion mri. Magnetic resonance in medicine 59, 1347–54. URL: http://europepmc.org/articles/PMC4667732/?report=abstract, doi:10.1002/mrm.21577.

Assaf, Y., Freidlin, R.Z., Rohde, G.K., Basser, P.J., 2004. New modeling and experimental framework to characterize hindered and restricted water diffusion in brain white matter. Magnetic resonance in medicine 52, 965–78. URL: http://www.ncbi.nlm.nih.gov/pubmed/15508168, doi:10.1002/mrm.20274.

Aydogan, D.B., Shi, Y., 2021. Parallel transport tractography. IEEE Transactions on Medical Imaging 40, 635–647. doi:10.1109/TMI.2020.3034038.

Baete, S.H., Cloos, M.A., Lin, Y.C., Placantonakis, D.G., Shepherd, T., Boada, F.E., 2019. Fingerprinting orientation distribution functions in diffusion mri detects smaller crossing angles. NeuroImage 198, 231–241. doi:10.1016/j.neuroimage.2019.05.024.

Baete, S.H., Yutzy, S., Boada, F.E., 2016. Radial q-space sampling for dsi. Magnetic Resonance in Medicine 76, 769–780. URL: https://onlinelibrary.wiley.com/doi/full/10.1002/mrm.25917https://onlinelibrary.wiley.com/doi/abs/10.1002/mrm.25917https:

//onlinelibrary.wiley.com/doi/10.1002/mrm.25917, doi:10.1002/MRM.25917/ASSET/SUPINFO/MRM25917-SUP-0001-SUPPINFO01.PDF.

Battocchio, M., Schiavi, S., Descoteaux, M., Daducci, A., 2020. Bundle-o-graphy.

Behrens, T.E.J., Woolrich, M.W., Jenkinson, M., Johansen-Berg, H., Nunes, R.G., Clare, S., Matthews, P.M., Brady, J.M., Smith, S.M., 2003. Characterization and propagation of uncertainty in diffusion-weighted mr imaging. Magnetic resonance in medicine 50, 1077–1088. URL: http://www.ncbi.nlm.nih.gov/pubmed/14587019, doi:10.1002/mrm.10609.

Canales-Rodríguez, E.J., Daducci, A., Sotiropoulos, S.N., Caruyer, E., Aja-Fernández, S., Radua, J., Mendizabal, J.M.Y., Iturria-Medina, Y., Melie-García, L., Alemán-Gómez, Y., Thiran, J.P., Sarró, S., Pomarol-Clotet, E., Salvador, R., 2015. Spherical deconvolution of multichannel diffusion mri data with non-gaussian noise models and spatial regularization. PLOS ONE 10, e0138910. URL: http://dx.plos.org/10.1371/journal.pone.0138910, doi:10.1371/journal.pone.0138910.

Cordero-Grande, L., Christiaens, D., Hutter, J., Price, A.N., Hajnal, J.V., 2019. Complex diffusion-weighted image estimation via matrix recovery under general noise models. NeuroImage 200, 391–404. doi:10.1016/j.neuroimage.2019.06.039.

Coronado-Leija, R., Ramirez-Manzanares, A., Marroquin, J.L., 2017. Estimation of individual axon bundle properties by a multi-resolution discrete-search method. Medical Image Analysis 42, 26–43. doi:10.1016/J.MEDIA.2017.06.008.

Daducci, A., Palu, A.D., Lemkaddem, A., Thiran, J.P., 2014. Commit: Convex optimization modeling for micro-structure informed tractography. IEEE Transactions on Medical Imaging 34.

Dell'Acqua, F., Lacerda, L.M., Catani, M., Simmons, A., 2013. Anisotropic power maps : A diffusion contrast to reveal low anisotropy tissues from hardi data.

Dhollander, T., Raffelt, D., Connelly, A., 2016. Unsupervised 3-tissue response function estimation from single-shell or multi-shell diffusion mr data without a co-registered t1 image.

Fick, R.H., Wassermann, D., Deriche, R., 2019. The dmipy toolbox: Diffusion mri multi-compartment modeling and microstructure recovery made easy. Frontiers in Neuroinformatics 13, 64. doi:10.3389/FNINF.2019.00064/BIBTEX.

Filipiak, P., Lin, Y.C., Placantonakis, D., Shepherd, T., Boada, F., Baete, S., 2021. Two methods to generate an odf-dictionary for odf-fingerprinting.

Frigo, M., Zucchelli, M., Deriche, R., Deslauriers-Gauthier, S., 2021. Talon: Tractograms as linear operators in neuroimaging.

Garyfallidis, E., Brett, M., Amirbekian, B., Rokem, A., Walt, S.V.D., Descoteaux, M., Nimmo-smith, I., Contributors, D., 2014. Dipy, a library for the analysis of diffusion mri data. Frontiers in Neuroinformatics 8, 1–5. URL: http://www.frontiersin.org/Journal/10.3389/fninf.2014.00008/abstract.

Garyfallidis, E., Brett, M., Correia, M.M., Williams, G.B., Nimmo-Smith, I., 2012. Quickbundles, a method for tractography simplification. Frontiers in neuroscience 6, 175. URL: http://www.frontiersin.org/Journal/10.3389/fnins.2012.00175/abstracthttp://journal.frontiersin.org/article/10.3389/fnins.2012.00175/abstract, doi:10.3389/fnins.2012.00175.

Girard, G., Whittingstall, K., Deriche, R., Descoteaux, M., 2014. Towards quantitative connectivity analysis: reducing tractography biases. NeuroImage 98, 266–278. URL: http://www.sciencedirect.com/science/article/pii/S1053811914003541, doi:10.1016/j.neuroimage.2014.04.074.

Gudbjartsson, H., Patz, S., 1995. The rician distribution of noisy mri data. Magnetic Resonance in Medicine 34, 910–914. doi:10.1002/mrm.1910340618.

Jeurissen, B., Tournier, J.D., Dhollander, T., Connelly, A., Sijbers, J., 2014. Multi-tissue constrained spherical deconvolution for improved analysis of multi-shell diffusion mri data. NeuroImage 103, 411–426. URL: http://www.sciencedirect.com/science/article/pii/S1053811914006442http://www.ncbi.nlm.nih.gov/

pubmed/25109526https://linkinghub.elsevier.com/retrieve/pii/S1053811914006442, doi:10.1016/j.neuroimage.2014.07.061.

Jordan, K.M., Amirbekian, B., Keshavan, A., Henry, R.G., 2018. Cluster confidence index: A streamline-wise pathway reproducibility metric for diffusion-weighted mri tractography. Journal of Neuroimaging 28, 64–69. URL: http://www.ncbi.nlm.nih.gov/pubmed/28940825http://doi.wiley.com/10.1111/jon.12467, doi:10.1111/jon.12467.

Kaden, E., Kelm, N.D., Carson, R.P., Does, M.D., Alexander, D.C., 2016. Multi-compartment microscopic diffusion imaging. NeuroImage 139, 346–359. doi:10.1016/j.neuroimage.2016.06.002.

Kellner, E., Dhital, B., Kiselev, V.G., Reisert, M., 2016. Gibbs-ringing artifact removal based on local subvoxel-shifts. Magnetic Resonance in Medicine 76, 1574–1581. URL: https://onlinelibrary.wiley.com/doi/full/10.1002/mrm.26054, doi:10.1002/MRM.26054.

Manjón, J.V., Coupé, P., Concha, L., Buades, A., Collins, D.L., Robles, M., 2013. Diffusion weighted image denoising using overcomplete local pca. PLoS ONE 8, e73021. doi:10.1371/journal.pone.0073021.

Ocampo-Pineda, M., Schiavi, S., Rheault, F., Girard, G., Petit, L., Descoteaux, M., Daducci, A., 2021. Hierarchical microstructure informed tractography. Brain Connectivity , brain.2020.0907URL: https://www.liebertpub.com/doi/10.1089/brain.2020.0907, doi:10.1089/brain.2020.0907.

Raffelt, D., Dhollander, T., Tournier, J.D., Tabbara, R., Smith, R., Pierre, E., Connelly, A., 2017. Bias field correction and intensity normalisation for quantitative analysis of apparent fibre density.

Sairanen, V., Ocampo-Pineda, M., Granziera, C., Schiavi, S., Daducci, A., 2021. Enhancing reliability of structural brain connectivity with outlier adjusted tractogram filtering, pp. 60–63.

Schiavi, S., Ocampo-Pineda, M., Barakovic, M., Petit, L., Descoteaux, M., Thiran, J.P., Daducci, A., 2020. A new method for accurate in vivo mapping of human brain connections using microstructural and anatomical information. Science Advances 6, eaba8245. URL: http://advances.sciencemag.org/, doi:10.1126/sciadv.aba8245.

Sedlar, S., Papadopoulo, T., Deriche, R., Deslauriers-Gauthier, S., 2021. Diffusion mri fiber orientation distribution function estimation using voxel-wise spherical u-net, Springer, Cham. pp. 95–106. doi:10.1007/978-3-030-73018-5_8.

Smith, R.E., Tournier, J.D., Calamante, F., Connelly, A., 2012. Anatomically-constrained tractography: Improved diffusion mri streamlines tractography through effective use of anatomical information. NeuroImage 63, 1924–1938. URL: http://www.ncbi.nlm.nih.gov/pubmed/22705374, doi:10.1016/j.neuroimage.2012.06.005.

Smith, R.E., Tournier, J.D., Calamante, F., Connelly, A., 2013. Sift: Spherical-deconvolution informed filtering of tractograms. NeuroImage 67, 298–312. URL: http://www.ncbi.nlm.nih.gov/pubmed/23238430, doi:10.1016/j.neuroimage.2012.11.049.

Smith, R.E., Tournier, J.D., Calamante, F., Connelly, A., 2015. Sift2: Enabling dense quantitative assessment of brain white matter connectivity using streamlines tractography. NeuroImage 119, 338–351. URL: http://www.sciencedirect.com/science/article/pii/S1053811915005972, doi:10.1016/j.neuroimage.2015.06.092.

Szafer, A., Zhong, J., Gore, J.C., 1995. Theoretical model for water diffusion in tissues. Magnetic Resonance in Medicine 33, 697–712. doi:10.1002/mrm.1910330516.

Tax, C.M., Jeurissen, B., Vos, S.B., Viergever, M.A., Leemans, A., 2014. Recursive calibration of the fiber response function for spherical deconvolution of diffusion mri data. NeuroImage 86, 67–80. URL: http://www.sciencedirect.com/science/article/pii/S1053811913008367.

Tournier, J.D., Calamante, F., Connelly, A., 2007. Robust determination of the fibre orientation distribution in diffusion mri: non-negativity constrained super-resolved spherical deconvolution. NeuroImage 35, 1459–1472. URL: http://www.ncbi.nlm.nih.gov/pubmed/17379540.

Tournier, J.D., Calamante, F., Connelly, A., 2010. Improved probabilistic streamlines tractography by 2nd order integration over fibre orientation distributions.

Tournier, J.D., Calamante, F., Connelly, A., 2013. A robust spherical deconvolution method for the analysis of low snr or low angular resolution diffusion data.

Tournier, J.D., Calamante, F., Gadian, D.G., Connelly, A., 2004. Direct estimation of the fiber orientation density function from diffusion-weighted mri data using spherical deconvolution. NeuroImage 23, 1176–85. URL: http://www.ncbi.nlm.nih.gov/pubmed/15528117, doi:10.1016/j.neuroimage.2004.07.037.

Tournier, J.D., Smith, R., Raffelt, D., Tabbara, R., Dhollander, T., Pietsch, M., Christiaens, D., Jeurissen, B., Yeh, C.H., Connelly, A., 2019. Mrtrix3: A fast, flexible and open software framework for medical image processing and visualisation. NeuroImage 202, 116137. doi:10.1016/j.neuroimage.2019.116137.

Tran, G., Shi, Y., 2015. Fiber orientation and compartment parameter estimation from multi-shell diffusion imaging. IEEE Transactions on Medical Imaging 34, 2320–2332. doi:10.1109/TMI.2015.2430850.

Tustison, N.J., Avants, B.B., Cook, P.A., Zheng, Y., Egan, A., Yushkevich, P.A., Gee, J.C., 2010. N4itk: Improved n3 bias correction. IEEE Transactions on Medical Imaging 29, 1310–1320. doi:10.1109/TMI.2010.2046908.

Veraart, J., Novikov, D.S., Christiaens, D., Ades-aron, B., Sijbers, J., Fieremans, E., 2016. Denoising of diffusion mri using random matrix theory. NeuroImage 142, 394–406. doi:10.1016/j.neuroimage.2016.08.016.

Wu, Y., Ahmad, S., Huynh, K., Liu, S., Thung, K., Lin, W., Yap, P.T., 2021a. An automated processing pipeline for diffusion mri in the baby connectome project.

Wu, Y., Hong, W., Lin, W., P.-T, Y., 2020a. Automated identification of non-brain voxels for clean brain extraction using diffusion mri.

Wu, Y., Hong, Y., Ahmad, S., Chang, W.T., Lin, W., Shen, D., Yap, P.T., 2020b. Globally optimized super-resolution of diffusion mri data via fiber continuity. Medical image computing and computer-assisted intervention : MICCAI ... International Conference on Medical Image Computing and Computer-Assisted Intervention 12267, 260.

URL: /pmc/articles/PMC8562653//pmc/articles/PMC8562653/?report=abstracthttps://www.ncbi.nlm.nih.gov/pmc/articles/PMC8562653/, doi:10.1007/978-3-030-59728-3_26.

Wu, Y., Hong, Y., Ahmad, S., Chang, W.T., Lin, W., Shen, D., Yap, P.T., 2020c. Globally optimized super-resolution of diffusion mri data via fiber continuity.

Wu, Y., Hong, Y., Ahmad, S., Lin, W., Shen, D., Yap, P.T., 2020d. Tract dictionary learning for fast and robust recognition of fiber bundles.

Wu, Y., Hong, Y., Ahmad, S., Yap, P.T., 2021b. Active cortex tractography.

Wu, Y., Hong, Y., Feng, Y., Shen, D., Yap, P.T., 2020e. Mitigating gyral bias in cortical tractography via asymmetric fiber orientation distributions. Medical Image Analysis 59, 101543. doi:10.1016/j.media.2019.101543.

Wu, Y., Hong, Y., Lin, W., Yap, P.T., 2020f. Model-free, fast, and automated correction of diffusion gradient orientations.

Wu, Y., Lin, W., Shen, D., Yap, P.T., 2019. Asymmetry spectrum imaging for baby diffusion tractography. doi:10.1007/978-3-030-20351-1_24.

Yeh, F., 2017. Diffusion mri reconstruction in dsi studio. Advanced Biomedical MRI Lab, National Taiwan University Hospital. Available online at: http://dsi-studio. labsolver.org/Manual/Reconstruction .

Zhang, Y., Brady, M., Smith, S., 2001. Segmentation of brain mr images through a hidden markov random field model and the expectation-maximization algorithm. IEEE Transactions on Medical Imaging 20, 45–57. URL: http://www.ncbi.nlm.nih.gov/pubmed/11293691, doi:10.1109/42.906424.

Zou, H., 2006. The adaptive lasso and its oracle properties. Journal of the American Statistical Association 101, 1418–1429. doi:10.1198/016214506000000735.
